# Supplementary material for: Pathway Of Low Anterior Resection syndrome (LARS) relief after Surgery (POLARiS): protocol for an international, open-label, multi-arm, phase 3 randomised superiority trial within a cohort, with economic evaluation, process evaluation and qualitative sub-study, to explore the natural history of LARS and compare transanal irrigation and sacral neuromodulation to optimised conservative management for people with major LARS following a high or low anterior resection for colorectal cancer
Source: BMJ Open. 2025 Feb 3;15(2):e092612. doi: 10.1136/bmjopen-2024-092612 (PMC11800293; doi:10.1136/bmjopen-2024-092612)
Supplement: Supplementary file 4 [file bmjopen-2024-092612.reviewer_comments.pdf]

PEER REVIEW HISTORY

BMJ Open publishes all reviews undertaken for accepted manuscripts. Reviewers are asked to complete a checklist review form and are provided with free text boxes to elaborate on their assessment. These free text comments are reproduced below.

ARTICLE DETAILS

Title (Provisional)

A prospective, international, open-label, multi-arm, phase 3 randomised superiority trial within a cohort, with economic evaluation, process evaluation and qualitative sub-study, to explore the natural history of Low Anterior Resection Syndrome (LARS) and compare trans-anal irrigation and sacral neuromodulation to optimised conservative management for people with major LARS.

Authors

Croft, Julie; Farrow, Emily; Coxon-Meggy, Alexandra Harriet; Gordon, Katie; Corrigan, Neil; Mather , Hannah; Stocken, Deborah D; Dale, Megan; Chong, Huey Yi; White, Judith; Knight, Laura; Meggy, Alun; Lloydwin, Christina; Tan, Betty; Douglas, Ashley; Powell, Ralph; Hepburn, Julie; Jayne, David; Torkington, Jared; Warwick , Andrea; Ng, Kheng-Seong; Wilson, Kate; Knowles, Charles H; Quyn, Aaron; Cornish, Julie

---

VERSION 1 - REVIEW

**Reviewer**	**1**
**Name**	**Zheng, Meichun**
**Affiliation**	**Sun Yat-Sen University Collaborative Innovation Center for Cancer Medicine**
**Date**	**30-Sep-2024**
**COI**	**None**

---

1. Please provide more detailed measurement time and indicators in the abstract

2. Supplement RCT blinding method

3. Describe the content of Booklet

4. Supplement quality control

**Reviewer**	**2**
**Name**	**Park, Ji Won**

Affiliation Seoul National University Hospital

Date 03-Oct-2024

COI None

Low anterior resection syndrome (LARS) is a significant postoperative complication experienced by many patients following rectal surgery. However, its progression and optimal management strategies remain poorly understood. This clinical study addresses this gap by providing a well-designed, pragmatic approach to investigating LARS. To further enhance the study's rigor and clinical relevance, the following points should be addressed:

1. The methodology for optimized conservative management lacks specificity. While exhaustive details may not be feasible, a concise overview of the key components related to diet, medication, and pelvic floor rehabilitation within the optimized conservative management protocol would significantly enhance the study's reproducibility and clinical applicability.

2. The randomization process requires clarification. Specifically, it should be stated whether block randomization was employed to ensure balanced group allocation.

3. Given the significant impact of anastomosis level on LARS, consideration should be given to stratifying patients based on high anterior resection versus low anterior resection. This stratification could provide valuable insights into the effectiveness of interventions across different surgical subgroups.

4. The inclusion of an interim analysis plan should be addressed. If planned, details on how these results might influence decisions regarding trial continuation or early termination should be provided. This information is crucial for understanding the study's adaptive design and ethical considerations.

VERSION 1 - AUTHOR RESPONSE

Response to Comments

Please revise your title so that study settings are included.

Response: This has been amended to include the patient population.

Please consider revising the abstract.

Response: This has been amended to include measurement time, as well as secondary endpoints. We hope this also addresses the first point made by Reviewer One.

Please revise Strengths & Limitation section.

Response: This has been amended to remove points which would be included an aim or a novelty and include additional strengths of the study design.

Please provide an example of the participant consent form as a “Supplemental Material” file. This file should be cited in the main text where informed consent procedures are described.

Response: The patient consent form for the cohort study and RCT have now been included as Supplementary 1, and is cited in the Methods & Analysis, Recruitment section.

Please include, as a supplementary file, the questions used in the semi-structured interview (as part of the qualitative sub-study).

Response: The interview guide has now been included as Supplementary 2 & 3 and is cited in the Qualitative Sub-Study section.

We recommend that you include a copy of the SPIRIT checklist indicating the page/ line numbers of the manuscript where the relevant information can be found.

Response: The SPIRIT checklist has now been updated to include line numbers.

Supplement RCT blinding method

Response: We have added additional detail to explain that neither clinician or participants are blinded to the RCT treatment allocation.

Describe the content of booklet

Response: We have now described the key components of the Optimised Conservative Management pathway in detail. We hope that this also addresses the points made by Reviewer Two.

Supplement quality control

Response: Treatment in the trial will be delivered at the discretion of the treating clinicians. There is no central review or verification of endpoints planned.

The randomisation process requires clarification. Specifically, it should be stated whether block randomisation was employed to ensure balanced group allocation.

Response: Additional detail has been included in the Methods & Analysis, Randomisation section.

Given the significant impact of anastomosis level on LARS, consideration should be given to stratifying patients based on HAR vs LAR. The stratification could provide valuable insights into the effectiveness of interventions across surgical subgroups.

Response: Procedure (HAR or LAR) is one of the stratification factors. We have added this detail to the randomisation section so this is more clear.

The inclusion of an interim analysis plan should be addressed.

Response: We have added detail into the Data Analysis Plan to explain that no interim analysis is planned, however the DMEC will review the safety and ethics of the trial by reviewing interim data and making recommendations to the Trial Steering Committee.

VERSION 2 - REVIEW

**Reviewer**	**2**
**Name**	**Park, Ji Won**
**Affiliation**	**Seoul National University Hospital**
**Date**	**27-Nov-2024**
**COI**	

The revisions and explanations provided are clear. I have no further questions or concerns at this time.
